# Human Lung Adenocarcinoma-Derived Organoid Models for Drug Screening

**DOI:** 10.1016/j.isci.2020.101411

**Published:** 2020-07-25

**Authors:** Zhichao Li, Youhui Qian, Wujiao Li, Lisa Liu, Lei Yu, Xia Liu, Guodong Wu, Youyu Wang, Weibin Luo, Fuyuan Fang, Yuchen Liu, Fei Song, Zhiming Cai, Wei Chen, Weiren Huang

**Affiliations:** 1 Shenzhen Second People's Hospital, The First Affiliated Hospital of Shenzhen University, Shenzhen, Guangdong 518035, China; 2 Shenzhen Institute of Synthetic Biology, Shenzhen Institutes of Advanced Technology, Chinese Academy of Sciences, Shenzhen, Guangdong 518055, China; 3 International Cancer Center, Shenzhen University School of Medicine, Shenzhen, Guangdong 518060, China; 4 The First Affiliated Hospital of Shantou University, Shantou, Guangdong 515041, China; 5 Guangdong Key Laboratory of Systems Biology and Synthetic Biology for Urogenital Tumors, Shenzhen, Guangdong 518035, China

**Keywords:** Tissue Engineering, Biotechnology, Cancer

## Abstract

Lung cancer is an extremely heterogeneous disease, and its treatment remains one of the most challenging tasks in medicine. Few existing laboratory lung cancer models can faithfully recapitulate the diversity of the disease and predict therapy response. Here, we establish 12 patient-derived organoids from the most common lung cancer subtype, lung adenocarcinoma (LADC). Extensive gene and histopathology profiling show that the tumor organoids retain the histological architectures, genomic landscapes, and gene expression profiles of their parental tumors. Patient-derived lung cancer organoids are amenable for biomarker identification and high-throughput drug screening *in vitro*. This study should enable the generation of patient-derived lung cancer organoid lines, which can be used to further the understanding of lung cancer pathophysiology and to assess drug response in personalized medicine.

## Highlights

• Generation of a living biobank of patient-derived lung adenocarcinoma organoids
• Organoid biobank encompasses most of known subtypes of adenocarcinoma
• Organoids maintain the histological and mutational spectrum of original tumors
• Tumor organoids provide a tool for biomarker identification and drug testing

## Graphical Abstract

**Figure:**
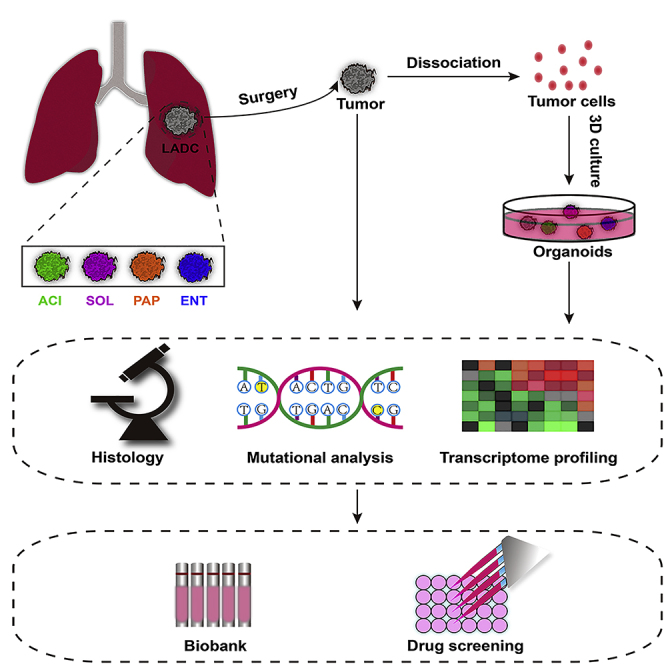

## Introduction

Lung cancer is the leading cause of cancer-related mortality globally. Every year, an estimated 1.82 million people are diagnosed with lung cancer and 1.56 million die from the disease (Ferlay et al., 2015). Lung cancer is divided into two main histological classes: non-small-cell lung cancer (NSCLC, ∼85%) and small-cell lung cancer (SCLC, ∼15%) (Herbst et al., 2008). There are two main histological subtypes of NSCLC: lung squamous-cell carcinoma (LSCC) and lung adenocarcinoma (LADC) (Molina et al., 2008). LADC has been the most predominant subtype of lung cancer since 1980s in the United States, and its incidence rates have been rising, whereas LSCC rates have been declining (Devesa et al., 2005; Patel et al., 2015).

Despite the considerable advances in the understanding of LADC, medical management is largely empirical and based on clinical and pathological features. However, owing to tumor heterogeneity among patients, clinical outcomes are usually unsatisfactory. The overall 5-year survival rate for lung cancer is 18% for all cancer stages combined in the United States (Siegel et al., 2018). Precision medicine, in which treatment regimens are individually tailored to each patient based on tumor characteristics, is believed to improve clinical outcomes. However, the development of precision medicine for LADC has been hampered by the lack of *in vitro* models in which the efficacy of candidate therapeutic regimens can be assessed.

The most commonly used LADC models are two-dimensional (2D) cell lines and patient-derived xenografts (PDXs). Although lung cancer cell lines, such as the LC2/ad cell line, PC-9 cell line, and VMRC-LCD cell line, have enabled advances in our understanding of lung cancer pathogenesis, they cannot fully recapitulate the three-dimensional (3D) structure of original tumors and do not retain the mutational profiles of their parental tumors (Suzuki et al., 2015). The PDX model retains the lung cancer mutational spectrum and the 3D organization, but the establishment of this model is inefficient and labor-intensive and usually takes several months per case, which makes it impractical to apply this model to guide precision medicine (Morgan et al., 2017; Moro et al., 2012). Therefore, there is an urgent necessity to establish a suitable model of LADC that faithfully recapitulates every aspect of its parental tumor and allows for large-scale drug screening.

Over the past few years, three-dimensional cancer organoid culture systems have been established from various cancers, including prostate cancer, colorectal cancer, pancreatic cancer, liver cancer, breast cancer, bladder cancer, and gastric cancer (Boj et al., 2015; Broutier et al., 2017; Gao et al., 2014; Lee et al., 2018; Sachs et al., 2018; van de Wetering et al., 2015; Yan et al., 2018). These cancer organoids have been shown to preserve the histological and mutational characteristics of their corresponding tumors and allow for drug screening. Several laboratories have established patient-derived lung cancer organoid lines from different histological and clinical stages as preclinical models for therapeutic drug screening (Kim et al., 2019; Li et al., 2020; Sachs et al., 2019; Shi et al., 2020).

In this study, we established 12 LADC organoid lines from tumor resections. In addition, we provided a thorough phenotypic and molecular characterization of LADC organoid lines and their parental tumors, including histological architecture, clinical marker expression, genomic landscape, and expression profile. Furthermore, we demonstrated the utility of LADC organoid lines as a model for drug testing to identify new therapeutic targets and advance personalized medicine. This study should be complementary to previous reports by providing a biobank of LADC organoids that covers a large spectrum of different LADC subtypes, including acinar (ACI), solid (SOL), papillary (PAP), and enteric (ENT) adenocarcinoma. We demonstrate that mechanical dissociation method is more suitable for the passage of LADC organoids. By comparing the transcriptome of LADC organoids with that of normal lung tissue-derived organoids, we identified several potential biomarkers associated with survival status when their expression levels were altered.

## Results

### Establishment of a Living Biobank of Patient-Derived LADC Organoids

**Figure 1:**
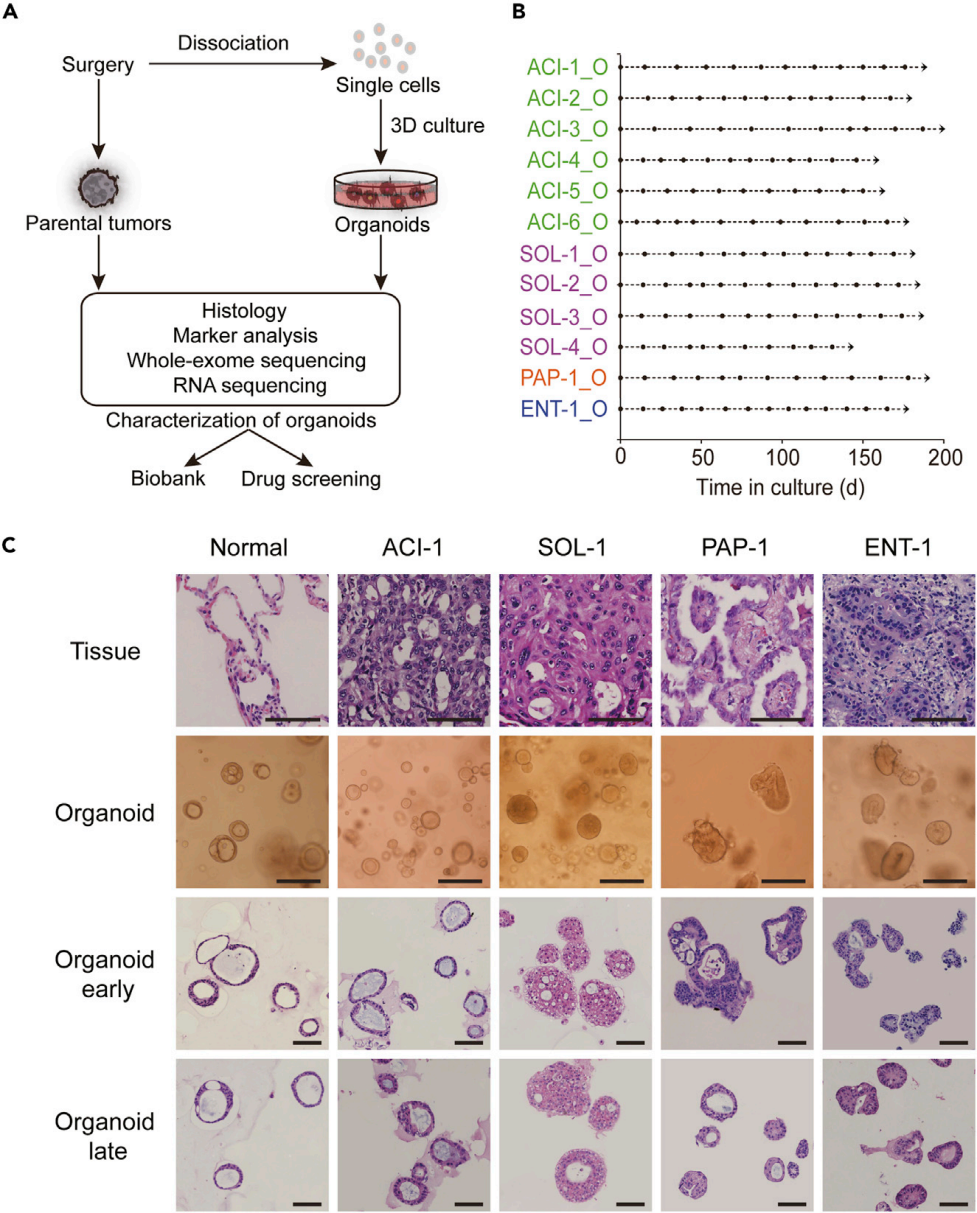
Establishment of Patient-Derived LADC Organoids (A) Overview of experimental design. (B) Expansion potential of LADC organoids established. Arrow, continuous expansion. Dot, passage. (C) Representative H&E staining of LADC tissues together with the bright-field images and H&E staining of the organoids derived from corresponding tumor tissues. Early passage (passage 5–10) and late passage (>10). Scale bar, 100 μm. See also Figure S2, Tables S1 and S2.

Surgically resected lung adenocarcinoma samples were obtained from untreated patients under informed consent. Each sample was split into four parts for organoid culture, histological analysis, genomic analysis, or transcriptomic analysis (Figure 1A). To isolate tumor cells, the resected tissue was minced with scissors and then digested with collagenase type II and trypsin. Tumor cells were plated in Matrigel drops and overlaid with organoid culture medium (Table S1). For the passaging of lung adenocarcinoma organoids, we first adopted the enzyme digestion protocol described in several reports (Li et al., 2018; Sachs et al., 2019). However, we noticed some lung adenocarcinoma organoid lines stopped growing after passaging (Figure S1). This problem was solved by using the mechanical dissociation method (see Transparent Methods). Organoid lines successfully maintained over five passages were regarded as success in organoid establishment. Using this protocol, we successfully established 12 human LADC-derived organoids from 15 patients with different subtypes of LADC (Table S2). The growth and passage of these LADC organoids were recorded (Figure 1B).

### LADC Organoids Preserve the Histological Structure of Parental Tumors

The majority of lung adenocarcinoma are classified into acinar (ACI), solid (SOL), papillary (PAP), micropapillary (MIP), or lepidic (LEP) subtypes based on their predominant histologic pattern (Eguchi et al., 2014; Mengoli et al., 2018; Travis et al., 2013).To test whether LADC organoids maintain the histological patterns present in the original patient samples, we performed hematoxylin and eosin (H&E) staining of paraffin sections from the organoids, as well as their corresponding parental tumors. The histological features of the parental tumors were highly recapitulated in the LADC organoid lines. For example, the LADC organoids from ACI adenocarcinoma presented a glandular and cystic-like structure, whereas the LADC organoids derived from SOL adenocarcinoma showed a solid and compact pattern (Figures 1C and S2). One LADC organoid line, ENT-1_O, established from an enteric adenocarcinoma sample, displayed a histological morphology similar to metastatic colorectal carcinoma, as observed in its corresponding parental tissue (Figure 1C). Compared with the normal lung organoids, which displayed a well-organized structure, LADC organoids always showed typical cancerous characteristics, such as enlarged nuclei and mucinous glands (Figures 1C and S2).

**Figure 2:**
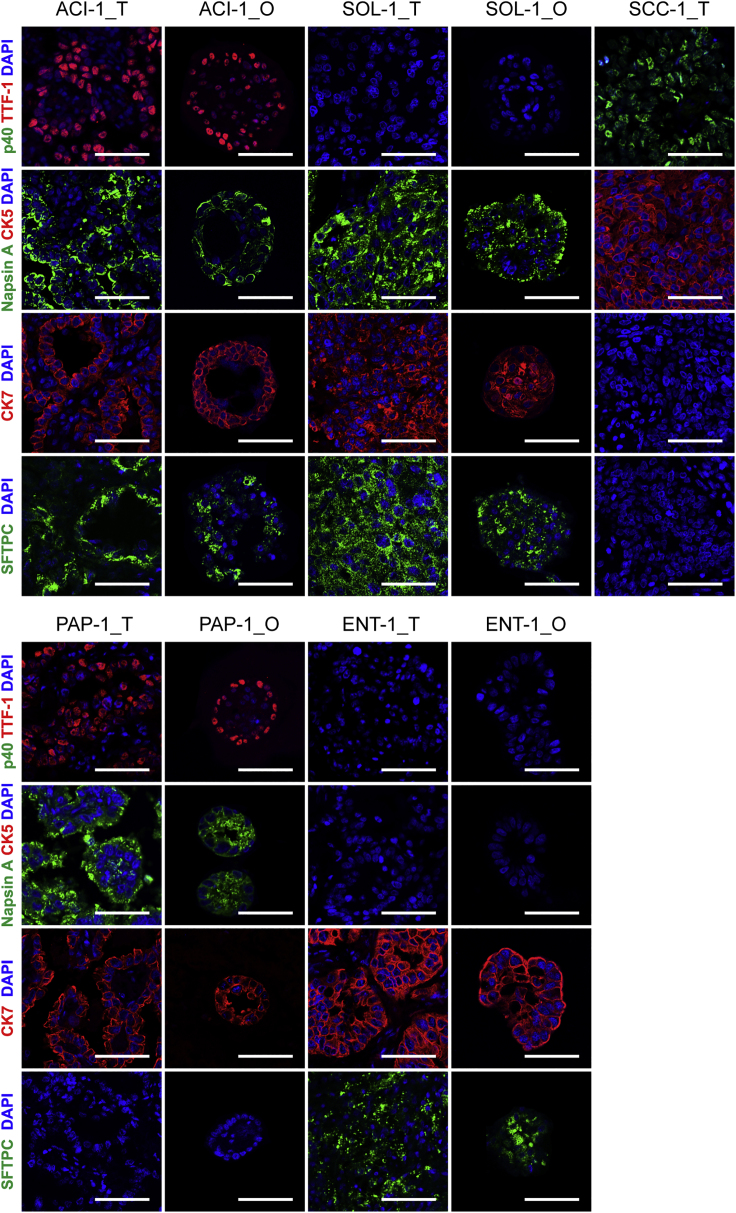
LADC-Derived Organoids Recapitulate Histopathological Characteristics of Parental Tumors Representative immunofluorescence images of paired LADC organoids (_O) and tumor tissues (_T) for TTF-1, Napsin A, p40, CK5, CK7, and SFTPC. Nuclei were stained with DAPI (blue). One lung squamous cell carcinoma sample, SCC-1_T, was also stained for these markers as a control. Scale bar, 100 μm. See also Figure S3 and Table S3.

To further characterize our LADC organoid lines, we performed immunofluorescence analysis of epithelial specific marker expression in each organoid line, as well as their corresponding parental tumors. We examined the expression of two adenocarcinoma markers, thyroid transcription factor (TTF-1) and Napsin A (Travis et al., 2010), as well as luminal epithelial marker cytokeratin 7 (CK7). All the organoid lines and their parental tumors were also stained for the most specific and sensitive squamous cell marker p40 (Tatsumori et al., 2014) and the basal epithelial marker cytokeratin 5 (CK5) to distinguish these LADC samples from LSCC samples. Most of the LADC organoid lines and the corresponding parental tumors exhibited positive staining of TTF-1 (n = 8/12) and Napsin A (n = 10/12), consistent with previous reports (Fatima et al., 2011; Gurda et al., 2015), and none of them were positive for CK5 and p40 (Figures 2 and S3). All the organoid lines displayed concordant marker expression profiles in the corresponding parental tumors. For example, TTF-1 was highly expressed in the ACI-1 and PAP-1 organoid lines and in their parental tissues but was absent in the SOL-1 and ENT-1 organoid lines and their parental tumors (Figure 2). Likewise, Napsin A was not expressed in the ENT-1 and ACI-3 organoid lines, in agreement with the expression pattern of their corresponding parental tumors, whereas the rest of the LADC organoid lines and their parental tumors were positive for Napsin A (Figures 2 and S3). All of the LADC organoid lines and their parental tumors expressed CK7, confirming an epithelial origin for all the organoids. Tumor samples and corresponding organoids also displayed a concordant expression pattern of Surfactant protein C (SFTPC), suggesting that alveolar type 2 (AT2) cells could be the cells of origin of these SFTPC^+^ samples (Figure 2).

In summary, these results demonstrate that the histological architecture and marker expression of the parental LADC tissues were recapitulated in the LADC organoids, even after long-term culture *in vitro*.

### LADC Organoid Lines Retain the Mutational Spectrum of the Parental Tumors

**Figure 3:**
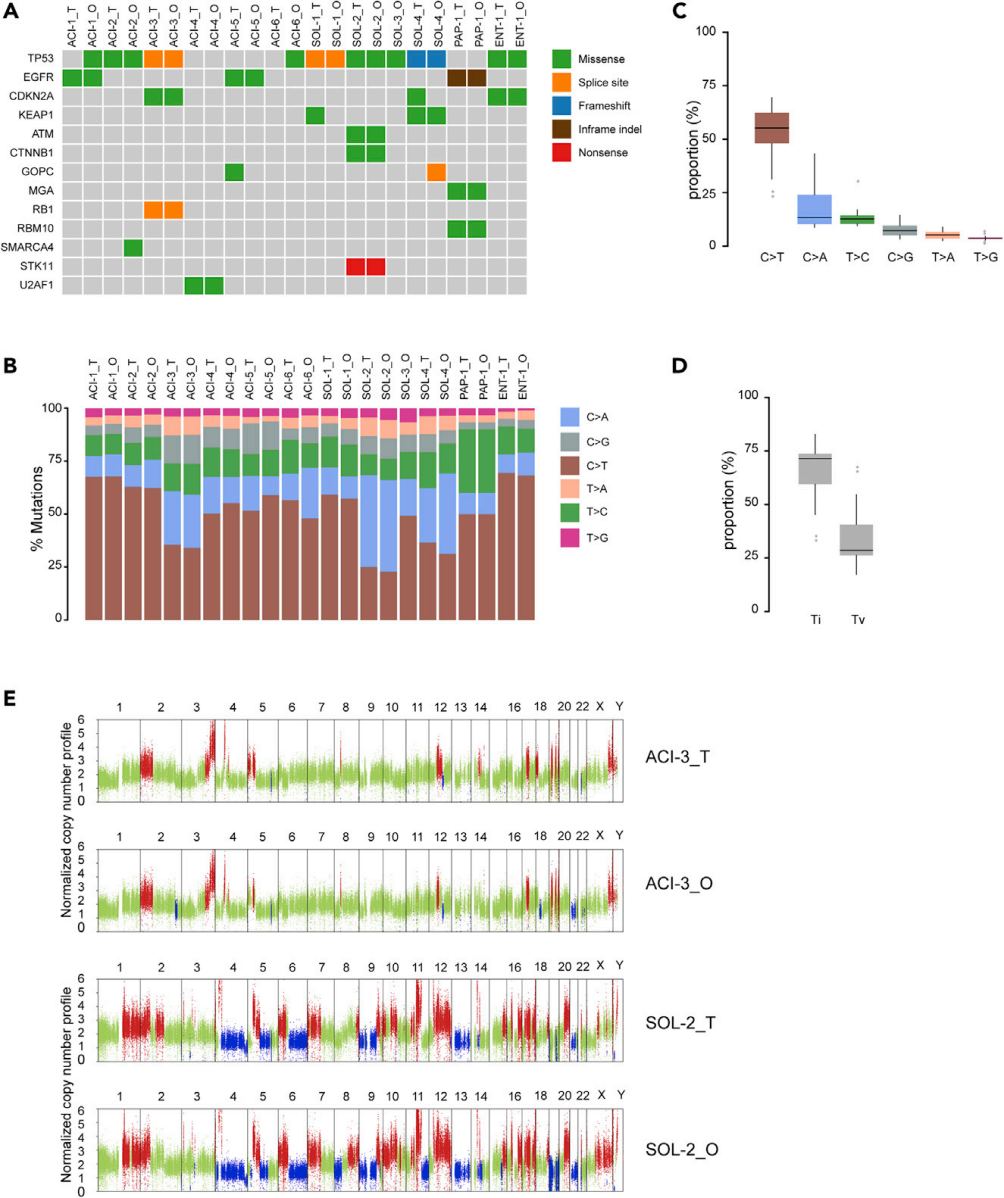
Mutational Signatures in LADC Organoid Lines (A) Summary of somatic mutations detected by deep targeted sequencing of LADC organoids (_O) and parental tumors (_T) where available. The type of mutation is indicated in the legend. (B) Proportions of exonic variants in LADC organoids (_O) and parental tumors (_T) and the six types of base substitutions are represented and indicated in the legend. (C) Percentage of the six types of base substitutions across all samples. Graph shown are mean ± SD. (D) Percentage of C>T/G>A transitions (Ti) and C>A/G>T transversions (Tv) across all samples. Graphs shown are mean ± SD. (E) Representative copy number landscape of paired LADC organoids (_O) and tumor tissues (_T). Red and blue represent DNA copy number gains and losses. See also Tables S4 and S5.

To determine whether the LADC organoid lines retain the genetic mutations present in the parental tumors, we performed whole-exome sequencing (WES) of the organoid lines and compared the results with those of the corresponding parental tumors. We filtered for variants and excluded polymorphisms present in organoid lines and parental tumors by comparing them to the analysis of matched patients' normal blood. The 12 LADC organoid lines displayed a heterogeneous set of cancer driver genes affected by missense mutations, splice site mutations, or frameshift mutation, some of which showed a variable pattern of alteration (Figure 3A). Comparative analysis showed that the somatic mutations present in the parental tumor tissue were highly retained in the corresponding LADC organoid lines. Importantly, the LADC organoid lines retained the majority of the most common genetic mutations in human LADC (Cancer Genome Atlas Research Network, 2014; Imielinski et al., 2012; Jordan et al., 2017). For example, *TP53*, the most commonly mutated gene in LADC, was mutated in most LADC organoid lines and their corresponding tumors (Figure 3A). We also identified cancer-associated mutated genes, including *EGFR*, *CDKN2A*, *KEAP1*, *ATM*, *CTNNB1*, *GOPC*, *MGA*, and *RB1*, almost all of which were conserved between organoid lines and parental tumors (Figure 3A; Table S4). We noticed that both the ACI-1_O and ACI-6_O lines harbored a *TP53* missense mutation, which is absent in their parental tumors (Figure 3A). This is likely to be explained by the pure tumor cellularity in the organoids or the intratumor diversification of the tumor samples.

To further determine the extent to which LADC organoid lines maintain the mutation spectrum of their parental tumors, we analyzed somatic substitutions in both tumor samples and organoids. The proportion of base substitutions was well retained among the LADC tissues and the corresponding cancer organoids (Figure 3B). Additionally, the most frequent base substitutions for both LADC tumor samples and organoids were C>T/G>A transitions (Ti) and C>A/G>T transversions (Tv), whereas the least frequent mutation type was T>G/A>C transversions (Figures 3B–3D), in agreement with the mutational spectrum described for LADC (Imielinski et al., 2012). Copy number variations (CNVs) analysis revealed similar patterns of DNA copy number gains and losses among LADC organoid lines and their corresponding tumors (Figure 3E; Table S5).

Intra-tumoral heterogeneity is a feature of LADC and may impact patient response to therapy. To assess whether intra-tumoral heterogeneity exists in LADC samples, we collected several parts of tumor tissues from one patient and sent them for whole-exome sequencing. Each tumor tissue was divided into three parts, and two tumor samples were collected. Genomic landscapes analysis revealed that subregion-specific genomic variants existed in different areas of the same tumor (Figure S4). For example, a *CCER1* missense mutation and a *CNTNAP3* missense mutation existed in the second part of sample ACI-7 but not in the other two subregions.

### LADC Organoids Recapitulate the Transcriptome of the Corresponding Parental Tumors

**Figure 4:**
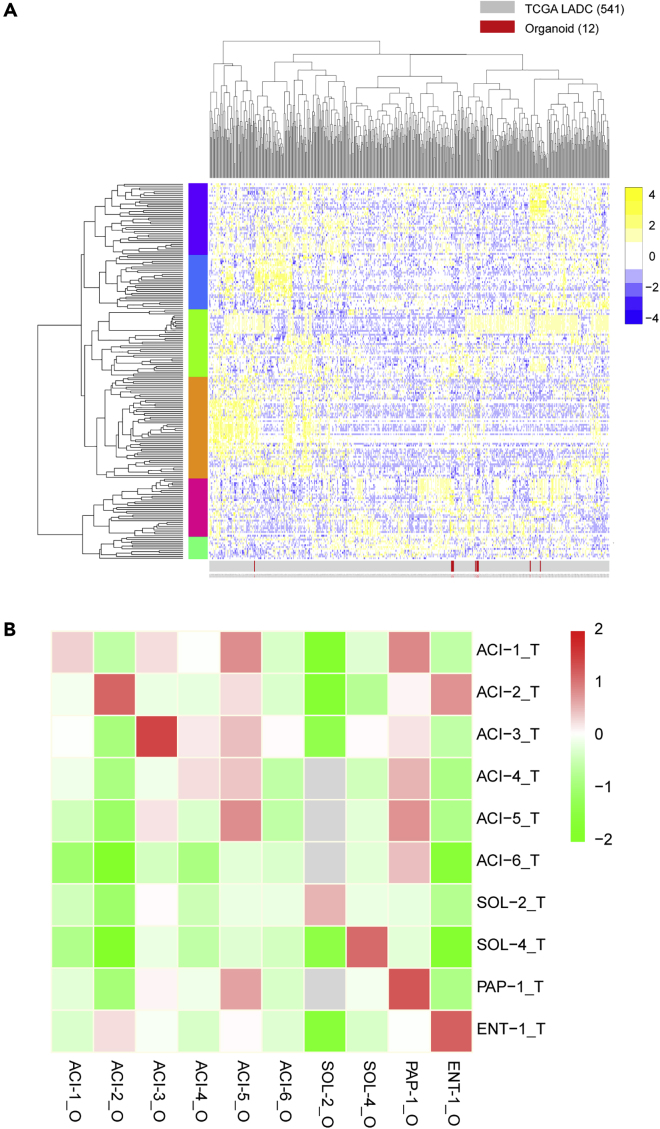
Global Gene Expression Analysis of LADC Organoids (A) RNA-seq data of 12 LADC organoid lines were normalized and combined with TCGA RNA-seq data (541 samples). The combined data were clustered using the 500 most-variable genes using 1-correlation distance with complete linkage. (B) Correlation heatmap of LADC organoids (_O) and tumor tissues (_T) based on RNA-seq expression data. Correlations were calculated for all paired organoids and tumors using all genes (column z-scored). See also Figure S5.

To further characterize our organoid lines, we performed RNA sequencing (RNA-seq) on LADC organoid lines and the corresponding tumor tissues. We compared the RNA-seq data of the LADC organoids with 541 LADC expression datasets from The Cancer Genome Atlas (TCGA) to determine whether our organoids were representative of the overall population of LADC. The results show that our organoids were distributed randomly throughout the dataset, suggesting representative gene expression profiles (Figure 4A). Gene expression correlation analysis showed that each LADC organoid line displayed a high concordance of expression profile to its corresponding tumor but not to any of the other tumor samples (Figure 4B). One exception was the ACI-6 line, the expression profile of which failed to highly correlate with its corresponding tumor, by failing to keep the lost *TP53* mutation seen in the tumor. In addition to global gene expression, we also evaluated the gene expression overlap in LADC-specific upregulated genes. These genes were identified by comparing the transcriptomes of LADC tumor samples with normal lung tissues from TCGA (>2-fold change, *p* < 0.05). The top 50 upregulated genes were selected in this study. The results showed that LADC organoids largely maintained the expression signatures of LADC-specific genes (Figure S5).

### LADC Organoids as a Model to Identify Potential Biom arkers

**Figure 5:**
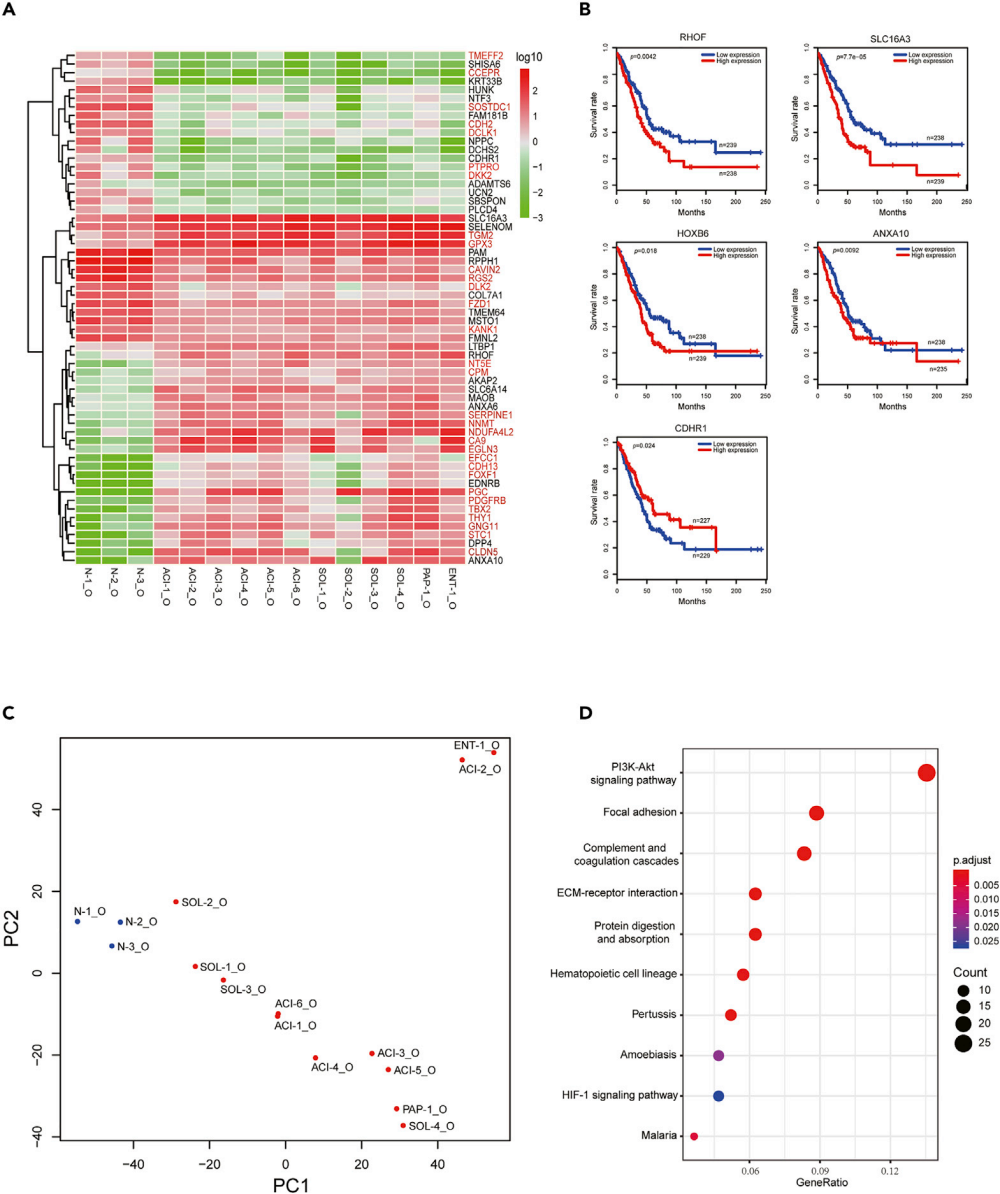
Use of LADC Organoids to Identify Tumor Biomarkers (A) Heatmap of 60 differentially expressed genes in LADC organoids, compared with normal lung organoids. Red indicates high expression; green indicates low expression. Genes marked in red were reported by others. (B) Kaplan-Meier survival analysis of patients with LADC based on the expression level of the indicated genes. (C) Principal components analysis (PCA) of RNA-seq data of all LADC organoids and normal lung organoids. (D) Significantly enriched KEGG signaling pathways in LADC organoids, compared with normal lung organoids.

In addition to LADC organoids, we also established three normal lung organoid lines (N-1_O, N-2_O, N-3_O) from normal wild-type lung samples. To explore the potential of LADC organoids as a model to identify tumor biomarkers, we compared the transcriptomes of all LADC organoid lines to those of all normal lung organoid lines. First, we performed principal component analysis (PCA) to determine the contribution of the cancerous state to the difference in gene expression between LADC organoids and normal lung organoid lines. The resulting data showed that normal lung organoids clustered together, whereas the LADC organoids were scattered all around, showing the tumor heterogeneity among patients (Figure 5C). Most LADC organoids belong to the same subtypes clustered together, with the exception of SOL-4_O line clustering with PAP-1_O and ACI-2_O clustering with ENT-1_O. PC1 component accounted for the variance between LADC organoids and normal lung organoids.

Next, we searched for differentially expressed genes between LADC organoids and normal lung organoids. Thirty upregulated genes and 30 downregulated genes with the lowest p values were selected for our analysis; we found that 20 genes had been reported to be upregulated and 11 genes had been reported to be downregulated in LADC, including *CA9*, *NT5E*, *EFCC1*, and *SERPINE1* (Figure 5A). We then studied the prognostic values of the remaining genes by performing survival analysis using publicly available TCGA databases. Within the 29 novel genes whose expression levels have never been reported to be altered in LADC samples, four genes were shown to be associated with survival status when their expression levels were altered (Figure 5B). *RHOF*, *SLC16A3*, and *ANXA10* predicted poor prognosis when overexpressed, whereas the downexpression of *CDHR1* correlated with poor overall survival (Figure 5B). Of one note was that *HOXB6* was also found to be upregulated in our LADC organoids and high expression was also associated with poor survival in patients with LADC. These data suggest that LADC organoids could be used as a model to identify tumor biomarkers.

We also performed KEGG (Kyoto Encyclopedia of Genes and Genomes) analysis to search for signaling pathways altered in LADC organoids. We found that the most enriched pathways in LADC organoids were the PI3K-Akt signaling pathway, pathways related to focal adhesion, complement and coagulation cascades, and the ECM-receptor interaction-associated pathways (Figure 5D).

### LADC Organoid Lines as a Model for Drug Screening

In order to evaluate the utility of lung adenocarcinoma organoids as a platform to predict patient-specific sensitivities to anticancer drugs, we performed high-throughput drug dose-response screens in 12 lung adenocarcinoma organoid lines. LADC organoid cultures were gently collected and plated in low-attachment 96-well plates in 2% Matrigel/growth medium. Organoids were treated with drugs 1 day after plating and incubated for 6 days before measuring the cell number using Cell Titer-Glo 3D reagent. For each lung adenocarcinoma organoid line, we tested its sensitivity to a library of 24 anti-cancer drugs using dose titration assays with technical replicates and biological triplicates (different passages of the same organoid line, between passages 5 and 10). Drugs were selected based on their clinical relevance for lung cancer treatment, including standard chemotherapy drugs and targeted agents against signaling pathways or molecules of interest. Drug sensitivity is shown by the half-maximal inhibitory concentration (IC_50_) and by the area under the dose-response curve (AUC).

**Figure 6:**
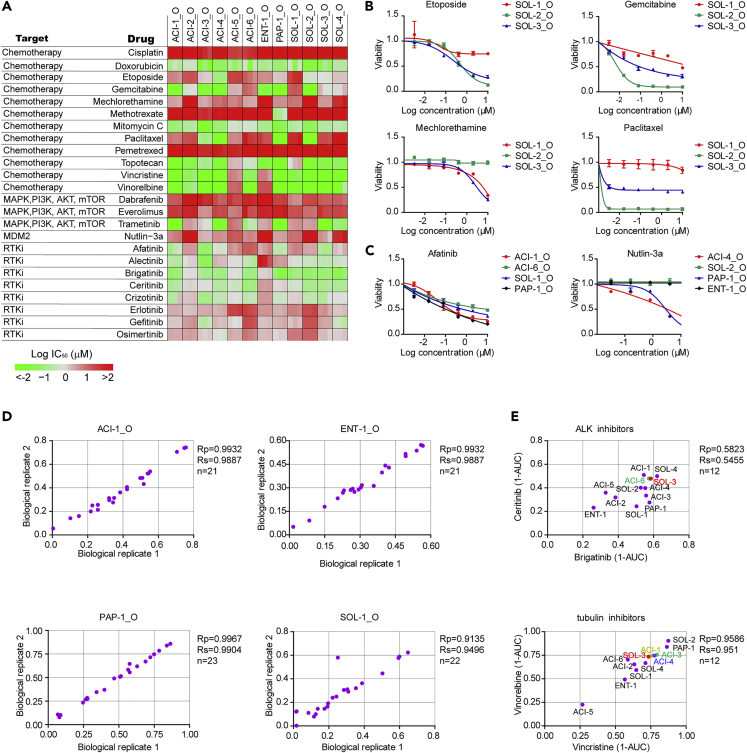
High-Throughput Drug Screening in LADC Organoids (A) Heatmap of logIC_50_ values for 24 compounds against LADC organoids by applying nonlinear regression (curve fit). (B and C) Dose-response curves of organoid lines to selected drugs. Each data point represents three biological replicates, with error bars representing ±SEM. (D) Representative scatterplots of 1-AUC (area under the curve) from two biological replicates of the drug screening data. Each data point is the 1-AUC for a drug used to treat the organoid line. (E) Representative scatterplots of 1-AUC from drug screening data of paired drugs with the same nominal targets.. See also Figure S6 and Table S6.

For each organoid line, its sensitivity to each particular drug was consistent between different passages, as revealed by the IC_50_ results and the high positive correlation of AUC values across biological replicates (Figures 6A and 6D). Individual lung adenocarcinoma organoid lines varied greatly in their responses to drugs owing to tumor heterogeneity. For example, the SOL-2_O organoid line was highly sensitive to gemcitabine, paclitaxel, and etoposide and resistant to mechlorethamine, whereas SOL-1_O line was sensitive to mechlorethamine and resistant to gemcitabine, paclitaxel, and etoposide (Figures 6A and 6B). Of particular interest was the PAP-1_O line with methotrexate, in which case we observed much higher sensitivity than that in all the rest of the LADC organoid lines (Figure 6A).

We also observed that drugs with similar nominal targets had comparable activity across all the LADC organoid lines. For example, the tubulin inhibitors vincristine and vinorelbine displayed similar sensitivity patterns and all the organoid lines showed a similar and concordant trend for the ALK-targeted drugs brigatinib and ceritinib (Figures 6A and 6E).

Some of the differences in responses to drugs between LADC organoid lines were in correlation with their mutational profiles. For example, the SOL-2_O line and ENT-1_O line displayed resistance to the p53-stablizing drug nutlin-3a, consistent with the presence of inactivating mutations in *TP53* (Vassilev et al., 2004) (Figure 6C). All the three organoid lines ACI-4_O, ACI-6_O, and PAP-1_O that were wild-type for *TP53* were sensitive to nutlin-3a (Figure S6). The organoid lines ACI-1_O and PAP-1_O demonstrated responses to the EGFR targeting agent afatinib, consistent with *EGFR* mutations in both samples (Figures 6A and 6C). Of note, the organoid lines ACI-3_O and SOL-3_O also demonstrated sensitivity to afatinib despite not having mutations in *EGFR* (Figure 6A), emphasizing the value of WES in combination with *in vitro* drug screens on LADC organoids.

We also observed a number of drugs with differential activities in the absence of an apparent genetic biomarker. For example, half of the LADC organoid lines were relatively more sensitive to trametinib, a MEK inhibitor, than the other half of the samples (Figure 6A). The ACI-3_O line and SOL-4_O line were sensitive to gefitinib, even though they did not harbor *EGFR* mutations (Figure 6A), highlighting the value of functional drug tests on LADC organoids.

## Discussion

In this study, we demonstrate the feasibility of generating cancer organoid lines from clinical LADC samples. A living biobank of 12 LADC organoid lines was generated from the most common LADC subtypes with a success rate of 80% (12/15). Compared with the conventional enzymatic digestion method, we demonstrate that the mechanical dissociation method in which the organoids are smashed into small fragments using 10-mL Stripette Serological Pipets is a better option for the passage of LADC organoids.

Comprehensive characterization of the LADC organoid lines confirm that they recapitulate the features of the corresponding parental tumors in terms of histological architecture, cancer driver gene mutations, copy number alterations (CNVs), single-nucleotide variants (SNV), and global gene expression profiles, even after long-term culture *in vitro*.

As LADC organoids preserve the genomic characteristics and global genes expression profiles of their corresponding parental tumors, we used LADC organoids as a model to identify tumor biomarkers. We found that the expression of *RHOF*, *SLC16A3*, *HOXB6*, *ANXA10*, and *CDHR1*, which was not reported to be altered in clinical LADC samples, was associated with survival status of patients with LADC and could be used as prognostic factors. The advantage of using LADC organoids over clinical LADC samples to search for tumor biomarkers is that LADC organoids are purer, so the differences in gene expression between tumor cells and nontumor cells are not affected by the presence of stromal cells and immune cells. Furthermore, the mechanism by which these tumor biomarkers affect tumor behaviors could be studied at the organoid level when combined with molecular biology techniques.

Lung cancer is generally thought to originate from the malignant transformation of adult lung stem cells (Wang et al., 2020; Zhang et al., 2015). LADC is believed to originate from alveolar type 2 (AT2) cells or their progenitors. *KRAS* (G12D) mutation in AT2 cells generates multifocal, clonal adenomas in transgenic mice (Desai et al., 2014). Another study demonstrates that CC10^+^ AT2 cells can give rise to LADC in response to *KRAS* (G12D) activation in mice (Xu et al., 2012). A recent study proves that the Sca-1^+^Abcg1^+^ bronchioalveolar epithelial cells are the cancer stem cell-like subset of AT2 cells and are the origin of LADC in *GPRC5A*-knockout mice (Yin et al., 2020). In addition to AT2 cells, Club cells are also shown to survive *KRAS* mutations and to form LADC after tobacco carcinogen exposure (Spella et al., 2019). Bronchoalveolar stem cells (BASCs) can proliferate *in vitro* and are expanded at early stages of tumorigenesis *in vivo* following *KRAS* (G12D) mutation, suggesting that BASCs may be the cell of origin for LADC (Kim et al., 2005).

Stage IV NSCLC accounts for approximately 40% of newly diagnosed lung cancer cases (Lemjabbar-Alaoui et al., 2015; Zappa and Mousa, 2016). Chemotherapy is one of the most common treatments for stage IV NSCLC. However, there are no reliable biomarkers for predicting its efficacy, which may be influenced by histology, age, and performance status. Targeted therapy, which works by specifically targeting molecular abnormalities present in the tumor cells, has proved to increase survival rate in patients with lung cancer (Camidge et al., 2012; Ou et al., 2016; Yang et al., 2015). Although the target gene mutations can be detected by WES, the patients' responses to targeted therapies were not always consistent with the expectations (Hirsch et al., 2017; Janne et al., 2015; Shaw et al., 2013). Thus, there is an urgent need to establish a suitable model to predict the patients' responses to chemotherapy and targeted therapy to increase the success rate.

Although suitable for high-throughput drug testing, traditional lung adenocarcinoma cell lines lack tissue architecture and cellular heterogeneity and are rarely of clinical relevance for individual patients. Patient-derived xenografts models of lung adenocarcinoma retain tumor histopathology and global gene expression of the patient's tumor but are resource intensive, time-consuming, and unsuitable for high-throughput drug screening. LADC organoid lines provide an opportunity to bridge the gap between traditional lung adenocarcinoma cell lines and patient-derived xenograft animal models. The LADC organoids-based high-throughput drug testing, in combination with the characterization of mutational profiles, could generate a link between lung LADC, genetics, and clinical trials to make personalized therapy designs and elucidate druggable targets.

Previous studies reported the use of lung cancer organoids to test drug responses (Kim et al., 2019; Sachs et al., 2019; Shi et al., 2020). In our study, LADC organoids were subjected to a larger library of anticancer drugs, including chemotherapeutic drugs and targeted drugs. We demonstrate that high-throughput drug screening is feasible in our LADC organoid biobank. Drug screening assays revealed striking differences in responses to a library of compounds between LADC organoid lines. There was a positive correlation of IC_50_ data and AUC value across biological replicates. The drugs with the same targets displayed reproducible sensitivity patterns among LADC organoid lines. We also observed a correlation between some drug sensitivities and mutational profiles.

Previous studies suggest that patient-derived gastrointestinal and gastric organoids could recapitulate patients' drug responses in the clinic (Vlachogiannis et al., 2018; Yan et al., 2018). As a next step, we will perform coclinical trials to determine whether the response of patient-derived LADC organoids to drugs *in vitro* recapitulates patients' responses to the same drugs *in vivo*.

### Limitations of the Study

High-throughput drug screening on organoids can facilitate personalized medicine and, when combined with WES and RNA-seq analysis, can contribute to the development of algorithms that accurately predict drug sensitivity. In this paper, this was hampered by the small sample size of patients. Collecting a larger number of LADC organoids would increase the statistical power to detect molecular markers of drug response.

### Resource Availability

#### Lead Contact

Correspondence and requests for materials and reagents should be directed to and will be fulfilled by the Lead Contact, Weiren Huang (pony8980@163.com).

#### Materials Availability

All organoid lines generated in this study will be available from the Lead Contact.

#### Data and Code Availability

The WES and transcriptome data generated during this study are available at Sequence Read Archive (SRA):SRR12059123-SRR12059164, SRR12072311-SRR12072333.

## Methods

All methods can be found in the accompanying Transparent Methods supplemental file.

## Supplemental Information

Document S1. Transparent Methods, Figures S1–S6, and Tables S1–S3 and S6

Table S4. Summary of Mutational Changes, Related to Figure 3

Table S5. Summary of CNVs, Related to Figure 3
