# Supplementary material for: Human Lung Adenocarcinoma-Derived Organoid Models for Drug Screening
Source: iScience. 2020 Jul 25;23(8):101411. doi: 10.1016/j.isci.2020.101411 (PMC7415928; doi:10.1016/j.isci.2020.101411)
Supplement: Document S1. Transparent Methods, Figures S1–S6, and Tables S1–S3 and S6 [file mmc1.pdf]

iScience, Volume *23*

Supplemental Information

Human Lung Adenocarcinoma-Derived Organoid Models for Drug Screening

Zhichao Li, Youhui Qian, Wujiao Li, Lisa Liu, Lei Yu, Xia Liu, Guodong Wu, Youyu Wang, Weibin Luo, Fuyuan Fang, Yuchen Liu, Fei Song, Zhiming Cai, Wei Chen, and Weiren Huang

Supplemental Information

Supplemental Figures

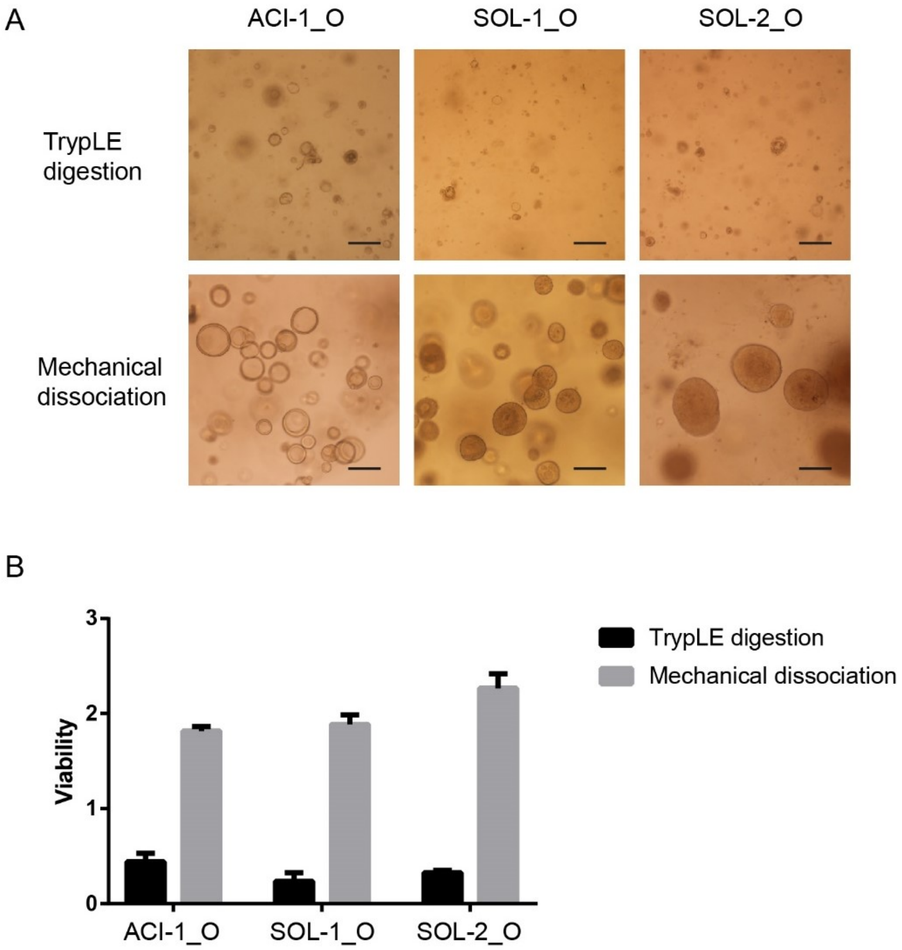

Figure S1. Comparative analysis of dissociation methods for the passage of LADC organoids, Related to Figure 1. (**A**) Representative bright-field images of organoids after passage using the indicated dissociation methods (14 days after passage). Scale bar, 100 μm. (**B**) Quantification of viable cells after passage using the indicated dissociation methods.

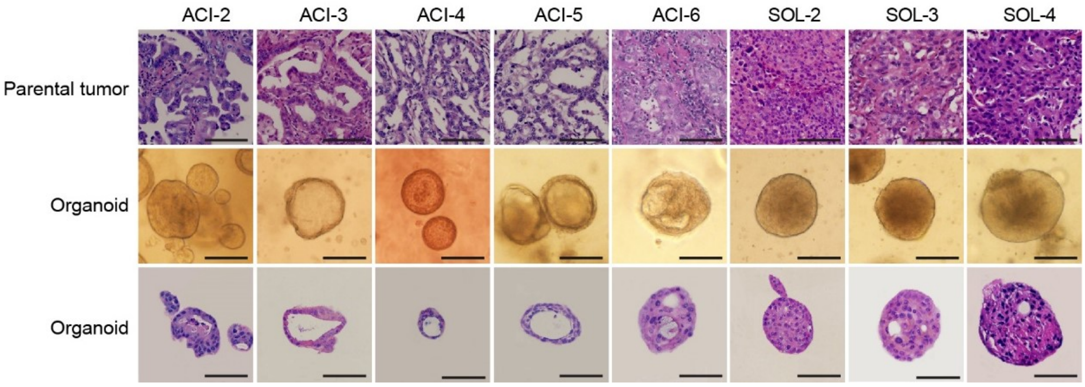

Figure S2. Histological analysis of LADC organoids, Related to Figure 1. H&E staining of LADC organoids and parental tumors, together with the bright-field images of LADC organoids (except for those that are shown in Figure 1C). Scale bar, 100 μm.

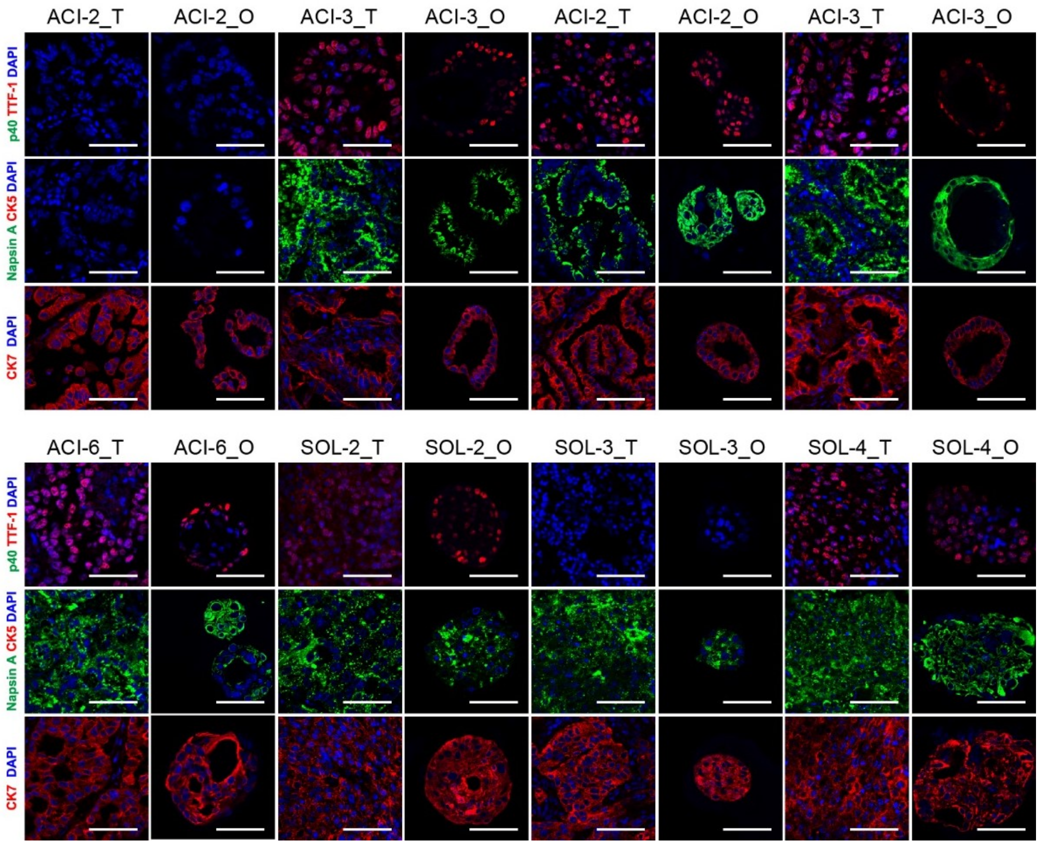

Figure S3. Immunofluorescence images of LADC organoids, Related to Figure 2. Shown above are the representative immunofluorescence images of the LADC organoids (_O) and their corresponding tumor tissues (_T) for p40, TTF-1, Napsin A, CK5, CK7 and DAPI. Scale bar, 100 μm.

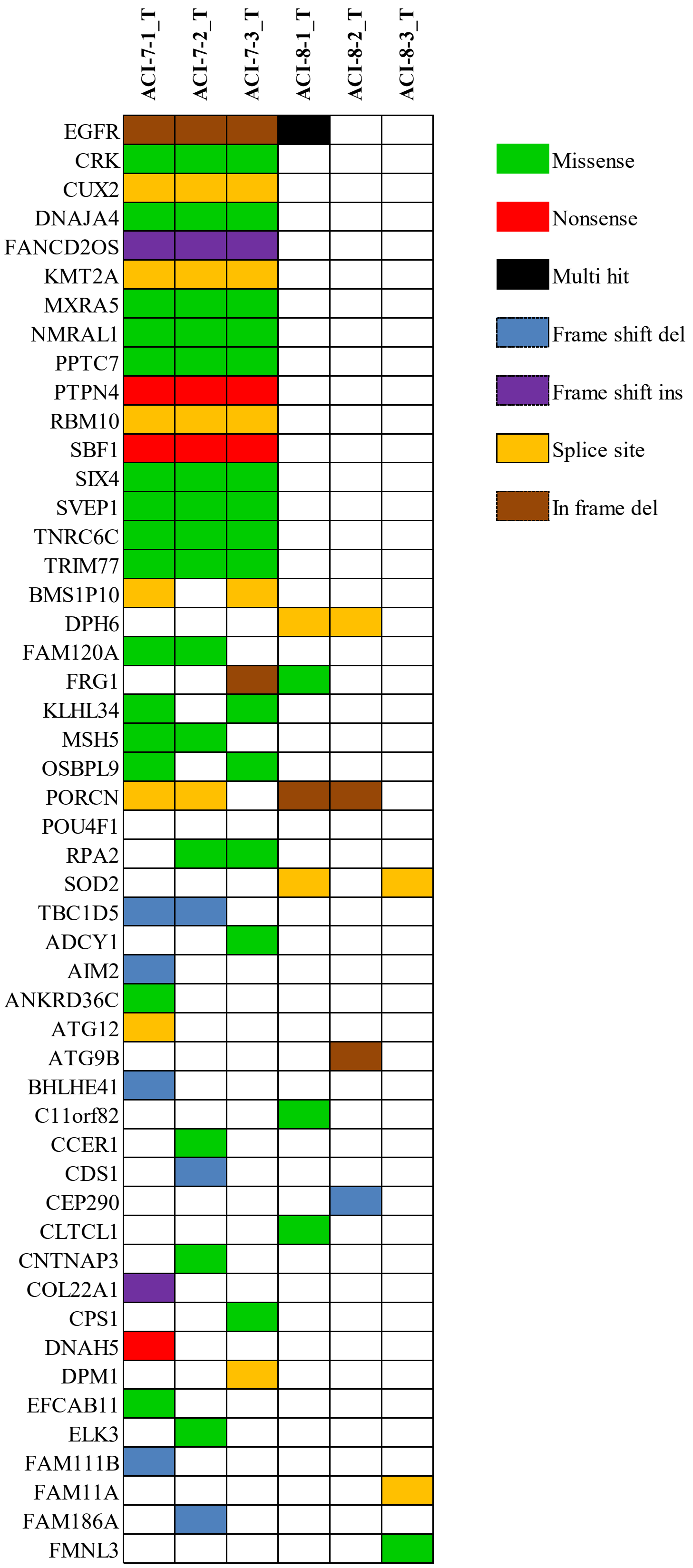

Figure S4. Mutational changes detected by deep targeted sequencing of subregion samples, Related to Figure 3. The type of mutations is indicated in the legend.

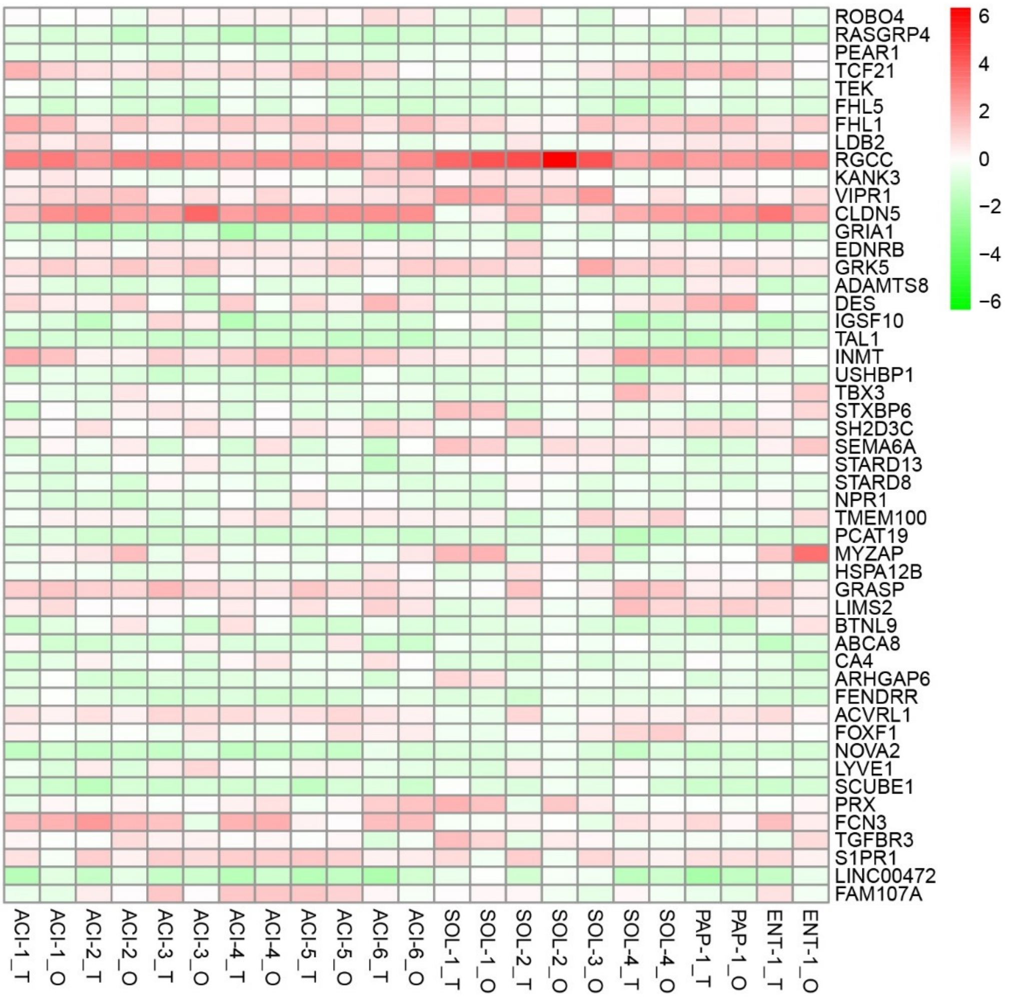

Figure S5. Gene expression heatmap of the top 50 LADC specific genes in LADC organoids (_O) and their parental tumors (_T), Related to Figure 4.

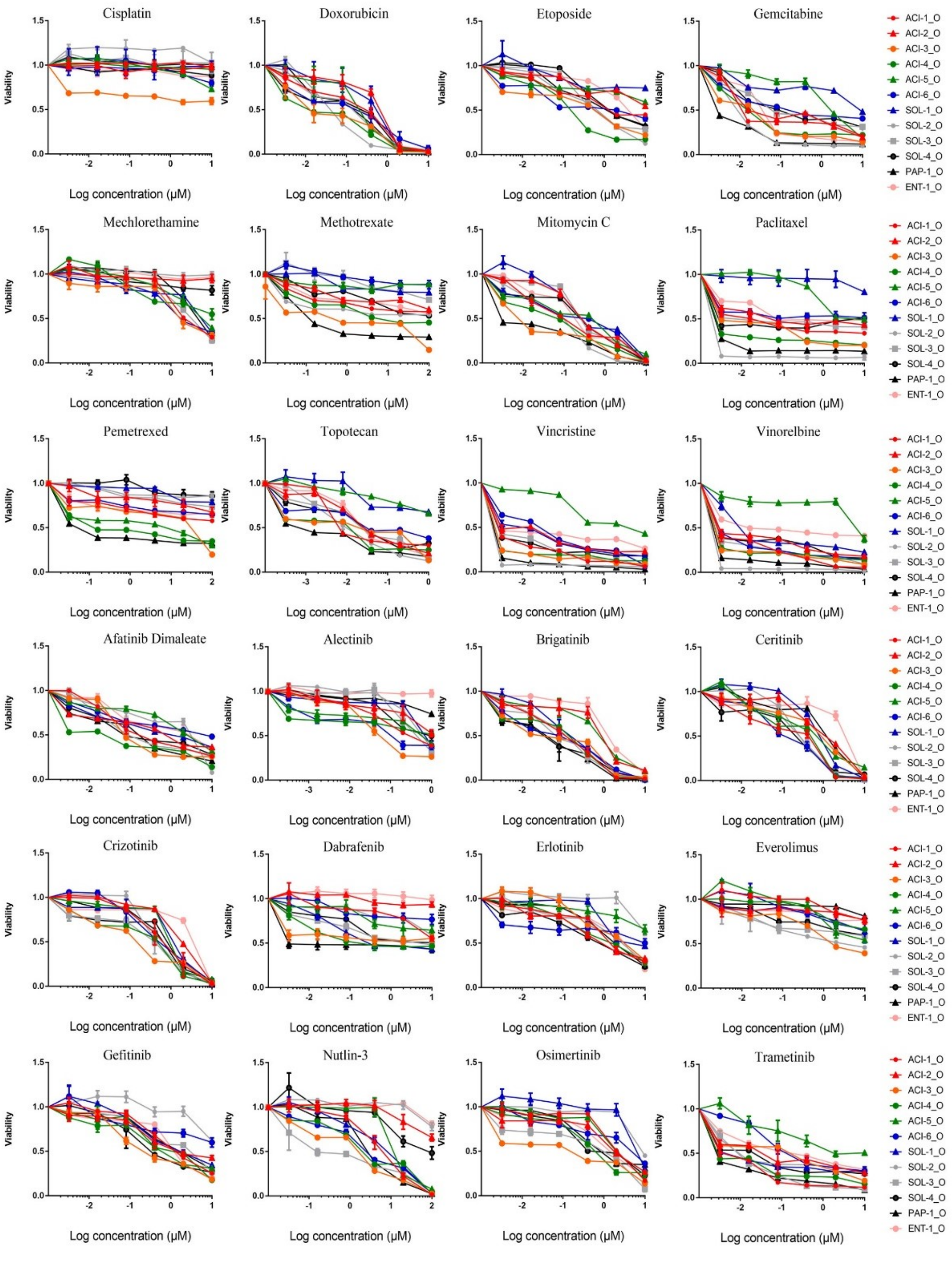

Figure S6. Dose response curves of LADC organoid lines to selected drugs, Related to Figure 6.

Supplemental Tables

Table S1. LADC organoid media recipe, Related to Figure 1.

Reagent Name	Supplier	Catalogue number	Final Concentration in Media
Advanced DMEM/F-12	Thermo Fisher Scientific	12634-010	1x
Antibiotic-Antimycotic (100X)	Thermo Fisher Scientific	15240-062	1x
GlutaMAX™ Supplement	Thermo Fisher Scientific	35050-061	1x
HEPES	Thermo Fisher Scientific	15630-080	10 mM
B-27™ Supplement (50X), serum free	Gibco	17504-044	1x
N-Acetylcysteine	Sigma	A9165	1.25 mM
Nicotinamide	Sigma	N0636	10 mM
SB202190	Sigma	S7076	10 mM
A83-01	Sigma	SML0788	500 nM
Recombinant Human R-Spondin 1 Protein	R&D Systems	4645-RS-250	500 ng/mL
Human Noggin	Peprotech	120-10C-1000	100 ng/mL
Y-27632	Abmole Bioscience	M1817	10 mM
Recombinant Human FGF-10	Peprotech	100-26	20 ng/mL
Recombinant Human KGF (FGF-7)	Peprotech	100-19	25 ng/mL

Table S2. Patients' information, Related to Figure 1.

Line	Diagnosis	Overall tumor stage	Metastasis	Gender	Age	Smoking
ACI-1_O	ACI	II	No	F	68	No
ACI-2_O	ACI	II	No	F	74	No
ACI-3_O	ACI	II	Yes, nodal metastasis	M	77	No
ACI-4_O	ACI	II	Yes, nodal metastasis	F	69	No
ACI-5_O	ACI	I	No	F	66	No
ACI-6_O	ACI	I	No	M	70	Yes
ACI-7	ACI	I	NO	F	66	No
ACI-8	ACI	II	NO	M	55	No
SOL-1_O	SOL	III	No	M	43	No
SOL-2_O	SOL	III	No	M	46	Yes
SOL-3_O	SOL	III	Yes, pleural metastasis	M	49	No
SOL-4_O	SOL	III	No	F	60	No
PAP-1_O	PAP	I	No	F	64	No
ENT-1_O	ENT	II	Yes, nodal metastasis	M	47	Yes

M: male, F: female

Line	Passage number for WES	Passage number for RNA seq	Passage number for HE staining and IF	Passage number for drug screening replicate 1	Passage number for drug screening replicate 2	Passage number for drug screening replicate 3
ACI-1_O	6	6	Early: 6; Late: 11	5	7	10
ACI-2_O	7	7	6	5	8	9
ACI-3_O	7	7	8	5	7	9
ACI-4_O	6	6	7	5	7	9
ACI-5_O	6	6	7	6	7	9
ACI-6_O	7	7	8	6	8	10

SOL-1_O	6	6	Early: 6; Late: 12	6	8	10
SOL-2_O	6	6	6	5	7	9
SOL-3_O	6	6	6	6	8	9
SOL-4_O	6	6	7	6	7	9
PAP-1_O	7	7	Early: 8; Late: 11	6	8	10
ENT-1_O	6	6	Early: 6; Late: 13	6	8	10

Table S3. Antibody information, Related to Figure 2.

Primary antibodies	Antigen	Supplier	Catalogue number	Origin	Dilution
	p40	Maixin Biotech	RAB-0666	rabbit	1/100
	TTF1	Maixin Biotech	MAB-0677	mouse	1/100
	Napsin A	Maixin Biotech	MAB-0704	mouse	1/100
	CK5	Thermo Fisher	RM-2106-S0	rabbit	1/100
	CK7	Thermo Fisher	180234	mouse	1/200
	SFTPC	Thermo Fisher	PA5-71680	rabbit	1/100
Secondary antibodies	Reagent Name	Supplier	Catalogue number	Origin	Dilution
	Donkey anti-Rabbit IgG (H+L) Highly Cross Adsorbed Secondary Antibody, Alexa Fluor 488	Thermo Fisher	A21206	donkey	1/1000
	Goat anti-Mouse IgG (H+L) Cross-Adsorbed Secondary Antibody, Alexa Fluor 555	Thermo Fisher	A21422	goat	1/1000

Table S6. List of drugs screened, Related to Figure 6.

Drug Name	Supplier	Catalogue number	Target	Max Concentration (µM)
Afatinib	Selleck Chemicals	S7810	EGFR/HER2	10 µM
Alectinib	Selleck Chemicals	S2762	ALK	1 µM
Brigatinib	Selleck Chemicals	S8229	ALK	10 µM
Ceritinib	Selleck Chemicals	S7083	ALK	10 µM
Cisplatin	Sigma	1134357	DNA crosslinker	10 µM
Crizotinib	Selleck Chemicals	S1068	ALK, c-Met	10 µM
Dabrafenib	Selleck Chemicals	S2807	BRAF	10 µM
Doxorubicin	Sigma	PHR1789	DNA replication	10 µM
Erlotinib	Selleck Chemicals	S1023	EGFR	10 µM
Etoposide	Selleck Chemicals	S1225	Topoisomerase II	10 µM
Everolimus	Sigma	E068 CERILLIANT	mTOR	10 µM
Gefitinib	Selleck Chemicals	S1025	EGFR	10 µM
Gemcitabine	Sigma	1288463	DNA replication	10 µM
Mechlorethamine	Selleck Chemicals	S4252	DNA crosslinker	10 µM
Methotrexate	Sigma	M9929	DHFR	100 µM
Mitomycin C	Selleck Chemicals	S8146	DNA crosslinker	10 µM
Nutlin-3a	Selleck Chemicals	S1061	MDM2	100 µM
Osimertinib	Selleck Chemicals	S7297	EGFR	10 µM
Paclitaxel	Selleck Chemicals	S1150	Tubulin	10 µM
Pemetrexed	Selleck Chemicals	S7785	TS, DHFR, GARFT	100 µM

Topotecan	Selleck Chemicals	S1231	Topoisomerase I	1 μM
Tramatenib	Selleck Chemicals	S2673	MEK1/2	10 μM
Vincristine	Selleck Chemicals	S1241	Tubulin	10 μM
Vinorelbine	Selleck Chemicals	S4269	Tubulin	10 μM

Transparent Methods

Tumor Specimen Collection

Lung adenocarcinoma (LADC) samples (1-4 cm^3) were obtained from the Second People's Hospital of Shenzhen and were transported directly to the laboratory after the surgery was performed. All patients provided informed consent. The study was approved by the Ethics Committee of the First Affiliated Hospital of Shenzhen University. Please see Table S2 for the clinical details of these samples.

Organoid Culture

Tumor samples were washed twice with cold PBS before minced into smaller pieces using scissors. A small piece was fixed in formalin for histopathological analysis and immunofluorescence. Half of the minced samples were frozen and stored in -80°C for DNA and RNA isolation, while the other half was used for the isolation of cancer cells. The minced tissue was incubated with collagenase II (5 mg/mL) with ROCK inhibitor Y-27632 dihydrochloride (10 μM) for 1 h at 37 °C. Digested tissues were spun down at 200 g for 5 min, washed once with AdDMEM/F12, and spun down again. The tissues were suspended in 5 mL of TrypLE Express with Y-27632 dihydrochloride (10 μM) for ~10 min at 37 °C. Trypsinization was stopped by the addition of 10 mL of AdDMEM/F12 supplemented with 1% penicillin/streptomycin, 1% Glutamax, 1% HEPES (Gibco), and 20% FBS. The dissociated cells were centrifuged at 200 g for 5 minutes and washed once with AdDMEM/F12 before being filtered through a 70-μM

cell strainer to remove large undigested clusters. The cells were centrifuged at 200 g for 5 minutes and resuspended with cold Matrigel. Six drops of 50-μL Matrigel-cell mixture (~20,000 cells/drop) were plated into one well of a 6-well plate. The drops were solidified for 2 minutes right side up and for another 8 minutes upside down at 37ºC and 5% CO_2. After the drops solidified, 2.5 mL of LADC organoid medium was added to each well. See Table S1 for the composition of the LADC organoid medium. The medium was replaced every 3 days and fresh medium was made weekly.

For passaging, Matrigel drops were scraped from the plate and centrifuged at 200 g for 3 minutes. The supernatant was discarded and the Matrigel was digested with TrypLE Express with Y-27632 dihydrochloride (10 μM) for ~5 min at 37 °C. The trypsinization was stopped by the addition of 10 mL of AdDMEM/F12 supplemented with 1% penicillin/streptomycin, 1% Glutamax, 1% HEPES, and 20% FBS. Organoids were centrifuged at 200 g for 5 min, washed once with AdDMEM/F12, and centrifuged again. Organoids (2-3 weeks after starting the culture) were dissociated into smaller clusters through trituration with 10 mL Stripette Serological Pipets. Organoids were passaged at a 1:2-1:3 ratio every 2-3 weeks.

To cryo-preserve organoids, organoids were dissociated from the Matrigel, and then frozen in recovery-cell-culture freezing medium (GIBCO) with Y-27632 dihydrochloride (10 μM).

Histology and Immunostaining

Tissues were washed in PBS and fixed and stored in 10% formalin before embedding and sectioning. Organoids were harvested and dissociated from the Matrigel, and fixed in 10% formalin.

Hematoxylin-eosin (H&E) staining and immunostaining were performed on 4 μm paraffin sections of cultured organoids and parental tumors. For H&E staining, paraffin sections were deparaffinized in xylene and rehydrated through a graded-ethanol series.

For immunofluorescence experiments, paraffin slides were subjected to antigen retrieval using EDTA solution (pH 8.0) after being deparaffinized and rehydrated. Sections were blocked in BSA blocking buffer (5% BSA in PBS) for 1 h at 37 °C and incubated with diluted primary antibodies (listed in Table S3) overnight at 4 °C. Slides were then washed with PBS and incubated with diluted secondary antibodies (listed in Table S3) for 1 h at room temperature. Nuclei were counterstained with DAPI. Immunofluorescence was imaged using a ZEISS confocal microscope.

Whole-Exome Sequencing and Analysis

The genomic DNA and RNA were isolated from primary tissue, blood, and organoids using the AllPrep DNA/RNA Mini Kit. Whole-Exome Sequencing (WES) libraries were generated using Agilent SureSelect Human All Exon V6 kit (Agilent Technologies, CA, USA) following manufacturer's recommendations and index codes were added to each sample. The WES libraries were sequenced with paired-end (2 X 150 bp) runs using Illumina X ten. The blood samples were sequenced to depths of 100 X (~12 Gb per sample) and tumor and organoids samples were ~200X (~24 Gb per sample). The sequencing data were filtered by adaptor and low quality reads by Fastp (v 0.12.6) (Chen et al., 2018). SNV were detected by GATK(v3.6) (McKenna et al., 2010). According to best practices guidelines, sequence reads were mapped against the human reference genome (UCSC hg19) using Burrows-Wheeler Alignment with maximal exact matches (BWA-MEM) v0.7.12 (Li and Durbin, 2009) followed by marked duplicates, merging of lanes, and realignment of INDELs. Base recalibration was not performed. Somatic SNVs and INDELs were determined by providing the reference (Blood) and tumor or organoid sequencing data to Mutect2 (options: -mbq 15 -allowPotentiallyMisencodedQuals) and Strelka2 (Kim et al., 2018) separately. Somatic SNVs with VAF<0.05 and supported by less than 3 reads were filtered out. CNAs were detected by Control-FREEC (v11.5) (Boeva et al., 2012) with BAM files. Mutation effect predictions and annotations were performed by Annovar (v) (Wang et al., 2010), COSMIC and dbSNP (Sherry et al., 2001).

RNA sequencing and analysis

RNA extraction was performed as described above. Sequencing libraries were generated using NEBNext® UltraTM RNA Library Prep Kit for Illumina® (NEB, USA) following the manufacturer's recommendations, and index codes were added to attribute sequences to each sample. The RNA libraries were sequenced with PE 150 bp using Illumina X ten. Low quality and adaptor polluted reads were removed. Then sequence reads were aligned to the reference genome (UCSC hg19) using STAR(v2.4.0j) (Dobin et al., 2013). Gene expression analysis was performed by RSEM (Li and Dewey, 2011), and differentially expressed genes were identified by EdgeR (Robinson et al., 2010).

We downloaded Level 3 RNA-seq LADC data from the TCGA Data Portal, and a total of 541 LADC samples' count data were used for analysis as provided. We normalized the expression data of the organoids and TCGA tumors together using ComBat (Leek et al., 2012), followed by normalization for sequencing depth and log10-transformation. Organoids and TCGA samples were clustered using the hclust function in R.

Principal components analysis (PCA) for normal lung tissue-derived organoids and LADC organoids were calculated by plotPCA function using the normalized EdgeR counts, the first two (PC1, PC2) were plotted. KEGG pathway enrichment of differentially expressed were performed by clusterProfiler.

We calculated differentially expressed genes between normal lung organoids and LADC organoids, then 30 upregulated genes and 30 downregulated genes in LADC organoids with the lowest *p*-value were listed. The survival analysis was performed using GEPIA server.

Organoid Drug Screening

To plate organoids for the drug screening, organoids were collected 5 days after passaging and filtered through a 100-μm cell strainer to remove large organoids. Collected organoids were resuspend in 2% Matrigel growth medium (20,000 organoids/mL) and dispensed into ultra-low attachment 96-well plates (100 μL/well) in triplicates. A 6-point 5-fold dilution series of 24 drugs (Listed in Table S6) were dispensed 24 h after plating. The maximal concentration of each drugs can be found in Table S6. Cell viability was assayed after 6 days of drug incubation using Cell Titer-Glo 3D (Promega) according to the manufacturer's instructions. Screens were performed in technical duplicates and biological triplicates.

Data analyses were performed using GraphPad Prism 7, the values of the IC_{50} and AUC were calculated by using nonlinear regression (curve fit) on the equation log (inhibitor) versus normalized response (variable slope).

Statistical Analysis

Statistical analyses were performed using GraphPad Prism 7. All summary data are presented as mean ± SEM, unless otherwise specified.

References

Boeva, V., Popova, T., Bleakley, K., Chiche, P., Cappo, J., Schleiermacher, G., Janoueix-Lerosey, I., Delattre, O., and Barillot, E. (2012). Control-FREEC: a tool for assessing copy number and allelic content using next-generation sequencing data. Bioinformatics (Oxford, England) 28, 423-425.

Chen, S., Zhou, Y., Chen, Y., and Gu, J. (2018). fastp: an ultra-fast all-in-one FASTQ preprocessor. Bioinformatics (Oxford, England) 34, i884-i890.

Dobin, A., Davis, C.A., Schlesinger, F., Drenkow, J., Zaleski, C., Jha, S., Batut, P., Chaisson, M., and Gingeras, T.R. (2013). STAR: ultrafast universal RNA-seq aligner. Bioinformatics (Oxford, England) 29, 15-21.

Kim, S., Scheffler, K., Halpern, A.L., Bekritsky, M.A., Noh, E., Kallberg, M., Chen, X., Kim, Y., Beyter, D., Krusche, P., et al. (2018). Strelka2: fast and accurate calling of germline and somatic variants. Nature methods 15, 591-594.

Leek, J.T., Johnson, W.E., Parker, H.S., Jaffe, A.E., and Storey, J.D. (2012). The sva package for removing batch effects and other unwanted variation in high-throughput experiments. Bioinformatics (Oxford, England) 28, 882-883.

Li, B., and Dewey, C.N. (2011). RSEM: accurate transcript quantification from RNA-Seq data with or without a reference genome. BMC bioinformatics 12, 323.

Li, H., and Durbin, R. (2009). Fast and accurate short read alignment with Burrows-Wheeler transform. Bioinformatics (Oxford, England) 25, 1754-1760.

McKenna, A., Hanna, M., Banks, E., Sivachenko, A., Cibulskis, K., Kernytsky, A., Garimella, K., Altshuler, D., Gabriel, S., Daly, M., et al. (2010). The Genome Analysis Toolkit: a MapReduce framework for analyzing next-generation DNA sequencing data. Genome research 20, 1297-1303.

Robinson, M.D., McCarthy, D.J., and Smyth, G.K. (2010). edgeR: a Bioconductor package for differential expression analysis of digital gene expression data. Bioinformatics (Oxford, England) 26, 139-140.

Sherry, S.T., Ward, M.H., Kholodov, M., Baker, J., Phan, L., Smigielski, E.M., and Sirotkin, K. (2001). dbSNP: the NCBI database of genetic variation. Nucleic acids research 29, 308-311.

Wang, K., Li, M., and Hakonarson, H. (2010). ANNOVAR: functional annotation of genetic variants from high-throughput sequencing data. Nucleic acids research 38, e164.
